# Isolation and Bioactivity of Secondary Metabolites from Solid Culture of the Fungus, *Alternaria sonchi*

**DOI:** 10.3390/biom10010081

**Published:** 2020-01-04

**Authors:** Anna Dalinova, Leonid Chisty, Dmitry Kochura, Varvara Garnyuk, Maria Petrova, Darya Prokofieva, Anton Yurchenko, Vsevolod Dubovik, Alexander Ivanov, Sergey Smirnov, Andrey Zolotarev, Alexander Berestetskiy

**Affiliations:** 1 All-Russian Institute of Plant Protection, Russian Academy of Agricultural Sciences, Pushkin, 196608 Saint-Petersburg, Russia; adalinova@vizr.spb.ru (A.D.); mar34915696@yandex.ru (M.P.); xasevak@gmail.com (V.D.); 2 Research Institute of Hygiene, Occupational Pathology and Human Ecology, Federal Medical Biological Agency, p/o Kuz’molovsky, 188663 Saint-Petersburg, Russia; mehrn.q2@gmail.com (L.C.); zcaryg@gmail.com (D.K.); vgar.spb@gmail.com (V.G.); darija-p1@yandex.ru (D.P.); 3 G.B. Elyakov Pacific Institute of Bioorganic Chemistry, Far Eastern Branch of Russian Academy of Sciences, 690022 Vladivostok, Russia; yurchant@ya.ru; 4 St. Petersburg State University, Universitetsky Av. 26, 198504 St. Petersburg, Russia; alexander.ivanov@spbu.ru (A.I.); sergey.smirnov@spbu.ru (S.S.); aazolotarev@gmail.com (A.Z.)

**Keywords:** *Alternaria sonchi*, perennial sowthistle, mycoherbicide, fungal xanthones, alternethanoxins, use of waste substrate

## Abstract

The fungus, *Alternaria sonchi* is considered to be a potential agent for the biocontrol of perennial sowthistle (*Sonchus arvensis*). A new chlorinated xanthone, methyl 8-hydroxy-3-methyl-4-chloro-9-oxo-9*H*-xanthene-1-carboxylate (**1**) and a new benzophenone derivative, 5-chloromoniliphenone (**2**), were isolated together with eleven structurally related compounds (**3**–**13**) from the solid culture of the fungus, which is used for the production of bioherbicidal inoculum of *A. sonchi*. Their structures were determined by spectroscopic (mostly by NMR and MS) methods. Alternethanoxins A and B, which were reported in *A. sonchi* earlier, were re-identified as moniliphenone and pinselin, respectively. The isolated compounds were tested for phytotoxic, antimicrobial, insecticidal, cytotoxic and esterase-inhibition activities. They did not demonstrate high phytotoxicity (lesions up to 2.5 mm in diameter/length at a concentration of 2 mg/mL) when tested on leaf disks/segments of perennial sowthistle (*Sonchus arvensis*) and couch grass (*Elytrigia repens*). They did not possess acute toxicity to *Paramecium caudatum*, and showed moderate to low cytotoxicity (IC_50_ > 25 µg/mL) for U937 and K562 tumor cell lines. However, chloromonilicin and methyl 3,8-dihydroxy-6-methyl-4-chloro-9-oxo-9*H*-xanthene-1-carboxylate (**4**) were shown to have antimicrobial properties with MIC 0.5–5 µg/disc. Compound **4** and chloromonilinic acid B were found to have contact insecticidal activity to wheat aphid (*Schizaphis graminum*) at 1 mg/mL. Compounds **2** and methyl 3,8-dihydroxy-6-methyl-9-oxo-9*H*-xanthene-1-carboxylate displayed selective carboxylesterase inhibition activity at concentration of 100 µg/mL. Therefore, the waste solid substrate for production of *A. sonchi* spores can be re-utilized for the isolation of a number of valuable natural products.

## 1. Introduction

Herbicides have revolutionized weed control over the last 65 years, but it also faces some challenges, including herbicide resistance in weeds, non-targeted adverse environmental effects, and soil and water pollution. Both organic and conventional agriculture need new effective and safe methods of weed control to avoid the problems related to intensive use of conventional chemical herbicides. For instance, the usage of biological and biochemical (natural or biorational) herbicides based on specific weed pathogens and natural products, respectively, is believed to assist the decreasing harmful impact of the chemicals [1].

Phytopathogenic fungi infecting weeds have been studied as producers of mycoherbicides, which are the plant protection products based on living fungal cells (mycelia or spores). The fungal inoculum is produced on artificial or natural substrates, after which it is formulated and applied in the same manner as chemical herbicides [2]. Moreover, phytotoxic metabolites of some necrotrophic phytopathogenic fungi have attracted interest as biochemical herbicides with new modes of action [3]. For instance, a tetramic acid derivative, macrocidin A, is known as the main phytotoxic component produced by *Phoma macrostoma*, a bioherbicide for the control of broadleaf weeds in golf turfgrasses. This phytotoxin affects the carotenoid biosynthesis by inhibiting phytoene desaturase [4,5,6].

There is the other side of the coin in the provisional use of mycoherbicides. Many fungi are known to produce very toxic compounds such as mycotoxins constraining the use of some effective but toxigenic mycoherbicidal agents (e.g., based on *Fusarium* spp.). Moreover, despite a number of registered mycoherbicides and a long list of potential ones [7], the toxicological risks of their application have been evaluated on limited number of fungal species such as *Stagonospora convolvuli*, *Ascochyta caulina*, *Myrothetium verrucarium* [8,9,10]. Recently, among phytotoxic compounds isolated from *S. cirsii*, a potential bioherbicide for *Sonchus arvensis* biocontrol, the most potent stagonolide A was considered as a mycotoxin due to a broad spectrum of biological activity [11,12]. Additionally, there is a general biotechnological problem of utilization of waste substrates [13] in the production of biopesticides. Depending on its composition and value, they can be re-used or destroyed.

Phytopathogenic fungi from the genus *Alternaria* are often considered as potential mycoherbicides [14,15,16,17] and producers of herbicidal molecules [3,18,19] as well as mycotoxins [20,21]. *Alternaria sonchi* J.J. Davis is a widely distributed leaf pathogen of sowthistles (*Sonchus* spp.) [22]. A strain of this fungus was recently patented as a candidate for biological control of perennial sowthistle in the Russian Federation. For this purpose, the fungus is grown on solid substrates (e.g., V8 agar medium or pearl barley) to produce conidia for field application and following induction of epidemics in the weed population [23].

**Figure 1:**
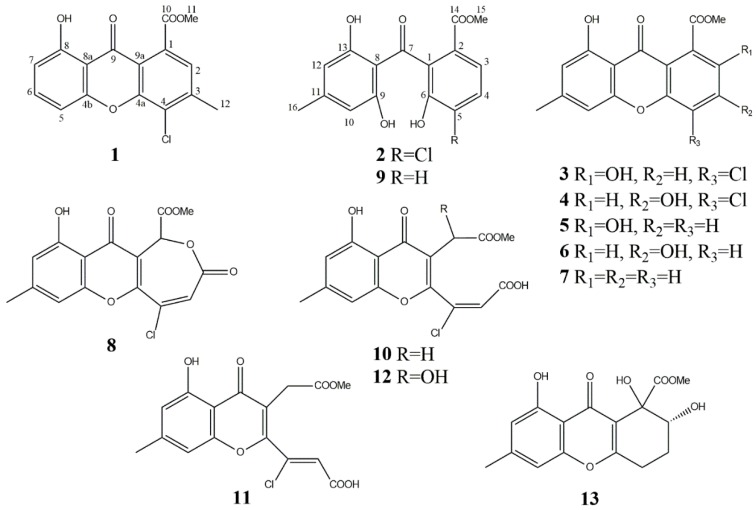
Structures of compounds **1**–**13** from *Alternaria sonchi*.

Five polycyclic ethanones (alternethanoxins A–E) and four chlorine-containing natural products (chloromonilicin, 4-chloropinselin, chloromonilinic acids B and C) were previously purified from solid-state cultures of *A. sonchi* S-102 on pearl barley (Figure 1) [24,25,26,27]. However, these compounds were poorly characterized toxicologically. In this paper, we report the isolation and wide biological characterization (phytotoxic, antimicrobial, insecticidal, cytotoxic, anti-esterase) of these and other secondary metabolites produced by *A. sonchi*, of which two compounds are new. Moreover, previously reported alternethanoxins A and B were re-identified as moniliphenone and pinselin, respectively. We discussed potential use of these metabolites in view of possible re-utilization of the waste solid substrate remained after production of *A. sonchi* conidia.

## 2. Materials and Methods

### 2.1. General Experimental Procedures

UV spectra were taken in MeCN solution on a Beckman Coulter DU800 spectrophotometer (Beckman Coulter, Fullerton, CA, USA). ^1^H and ^13^C NMR spectra were recorded at 400 and at 100 MHz, respectively, in CDCl_3_ as a solvent on a Bruker AVANCE III 400 MHz spectrometer (Bruker, Karlshrue, Germany). The solvent residual signal (δ 7.26 ppm) for ^1^H NMR spectra and the carbon signal of CDCl_3_ (δ 77.16 ppm) for ^13^C NMR spectra were used as references. Carbon multiplicities were determined by distortionless enhancement by polarization transfer (DEPT) spectra. DEPT, correlation spectroscopy (COSY)-45, heteronuclear multiple-quantum coherence (HSQC), and heteronuclear multiple-bond correlation (HMBC) were performed using standard Bruker microprograms [28]. ESI-MS spectra were recorded with TSQ Quantum Access spectrometer (Thermo Scientific, Waltham, MA, USA) after HPLC. High-resolution ESI-MS (HRESIMS) spectra were recorded on 6530 Accurate-Mass Q-TOF LC/MC spectrometer (Agilent Technologies, Santa Clara, CA, USA). Analytical and preparative TLC was performed on silica gel and reverse phase (Kieselgel 60 F254 plates, 0.25 and 0.5 mm, and Kieselgel 60 RP-18 F254 plates, 0.20 mm, respectively) (Merck, Darmstadt, Germany). The spots were visualized by exposure to UV radiation (254 nm) and/or by spraying with reagent anisaldehyde—H_2_SO_4_, followed by heating at 120 °C for 10 min. Column chromatography was performed on a silica gel column (Kieselgel 60, 0.063−0.200 mm) (Merck, Darmstadt, Germany). Medium-pressure chromatography (MPLC) was performed with a Sepacore chromatography system (Büchi, Flawil, Switzerand) using prepacked normal-phase (Silica HP 30 μm 40G and Silica HP 15 μm 12G) and reverse-phase (C18 HP 15 μm 20G) Puriflash columns (Interchim, Montluçon, France). Analytical HPLC was performed with Aquity UPLC H-Class (Waters, Milford, MA, USA), equipped with a photodiode array detector.

### 2.2. Production and Purification of Fungal Metabolites

The strain (S-102) of *A. sonchi* used in this study was deposited in the collection of All-Russian Research Institute of Plant Protection (Pushkin, Saint Petersburg, Russia). The fungus was grown on autoclaved pearl barley (150 g pearl barley and 100 mL deionized water per 1000 mL Erlemeyer flask) for 3 weeks at day temperature 24 °C, night temperature 20 °C and 12-h photoperiod. The fresh solid culture (4 kg) was extracted two times with 10 L hexane by shaking for 10 min. The combined hexane extracts were dried over anhydrous sodium sulfate and evaporated to dryness under vacuum at 40 °C to afford the crude hexane extract (4 g). Then, the dry fungal culture was blended and extracted repeatedly with 10 L of a mixture of Me_2_CO-H_2_O (50:50, *v*/*v*) according to the procedure previously reported [24]. After evaporation of the Me_2_CO, H_2_O phase was passed through a column containing 60 g of Diaion HP-20 (Sigma-Aldrich, St. Louis, MO, USA), and then, it was washed with water and eluted with MeOH. The extract was evaporated to dryness under vacuum at 40°C to afford the crude solid phase extract (12 g).

The crude hexane extract was separated on glass column packed with Sephadex LH-20 (Sigma-Aldrich, St. Louis, MO, USA) using a mixture of CH_2_Cl_2_-Me_2_CO (1:1, *v*/*v*) as the eluent. Resulting fractions HB (720 mg) and HE (280 mg) were chromatographed by MPLC on silica gel cartridge (Si-HP, 25 µm, 40 g, Interchim, Montluçon, France) with n-hexane-Me_2_CO gradient elution and then purified by preparative HPLC to afford **3** (100 mg), **5** (100 mg), and **8** (46 mg). Fractions HC (240 mg) and HD (105 mg) were chromatographed in the same conditions and gave **1** (25 mg) and **7** (52 mg).

The crude solid phase extract was fractionated by Toyopearl HW-40F (Tosoh Bioscience, Tokyo, Japan) column chromatography using MeOH as the eluent to afford eight fractions on the basis of TLC. The most phytotoxic fraction CM (6 g) was further separated by gel-chromatography on Toyopearl HW-40F eluted with a gradient of MeOH-water from 20:80 to 100:0. The fraction 5CM (1.5 g) was applied to a cartridge Chromabond C18ec (10 g, Macherey-Nagel, Düren, Germany), the column was eluted sequentially with 40 mL of MeOH-H_2_O (25:75, 50:50, 75:25, 100:0, *v*/*v*), yielding four subfractions. The second subfraction (700 mg) was separated by HPLC (MeCN-0.1% formic acid, 40:60, *v*/*v*) to afford **2** (30 mg), **10** (43 mg), **11** (30 mg), and **12** (11 mg). The third subfraction (150 mg) was further purified by HPLC (MeCN-0.1% formic acid, 60:40, *v*/*v*) to afford **4** (13 mg). Further purification of the fourth subfraction (100 mg) with HPLC (MeCN-0.1% formic acid, 30:70, *v*/*v*) gave **13** (48 mg). The fraction 7CM (1.5 g) was subjected to reverse-phase MPLC and eluted with MeCN-water (40:60 to 100:0, *v*/*v*) to give 10 subfractions. The seventh and the ninth subfractions were finally purified by HPLC to afford **6** (4 mg) and **9** (13 mg) respectively.

Methyl 8-hydroxy-3-methyl-4-chloro-9-oxo-9H-xanthene-1-carboxylate (**1**): UV (MeCN) λ_max_ (log *ε*) 235 (3.77), 255 (3.67), 288 sh. (3.21), 305 (3.22), 366 (2.99) nm; HRESIMS *m/z* 341.0172 [M + Na]^+^ (calcd for C_16_H_11_ClO_5_Na^+^, 341.0193); ^1^H and ^13^C NMR see Table 1.

5-Chloromoniliphenone (**2**): UV (MeCN) λ_max_ (log *ε*) 205 (4.48), 247 (3.97), 287 (4.04), 351 (3.36) nm; HRESIMS *m/z* 359.0282 [M + Na]^+^ (calcd for C_16_H_13_ClO_6_Na^+^, 359.0298); ^1^H and ^13^C NMR see Table 2.

Methyl 3,8-dihydroxy-6-methyl-9-oxo-9H-xanthene-1-carboxylate (**6**): UV (MeCN) λ_max_ (log *ε*) 234 (4.23), 251 (4.04), 267 sh. (3.83), 303 (3.92), 355 (3.60) nm; HRESIMS *m*/*z* 323.0521 [M + Na]^+^ (calcd for C_16_H_12_O_6_Na^+^, 323.0531); ^1^H (400 MHz, CDCl_3_) *δ* 12.36 (s, 1H), 6.87 (s, 1H), 6.83 (s, 1H), 6.68 (s, 1H), 6.59 (s, 1H), 4.00 (s, 3H), 2.40 (s, 3H). ^13^C (100 MHz, CDCl_3_) *δ* 179.7, 169.7, 163.4, 161.4, 158.0, 155.8, 148.3, 135.0, 112.9, 111.4, 110.4, 107.1, 106.5, 103.8, 53.1, 22.5.

### 2.3. X-ray Experimental

For a single crystal X-ray diffraction experiment, suitable crystals of **1** were fixed on a micro-mount. Diffraction data were collected using an XtaLab SuperNova diffractometer (Rigaku Oxford Diffraction, Oxford, UK) at a temperature of 100 K using monochromated CuKα radiation. The structure was solved by direct methods using the SHELX program incorporated in the OLEX^2^ crystallography software (OlexSys Ltd, Chemistry Department, Durham University, DH1 3LE, UK) [29,30]. The crystallographic data and some parameters of refinement are presented in Table S1. The carbon-bound H atoms were placed in calculated positions and were included in the refinement in the ‘riding’ model approximation. Empirical absorption correction was applied in CrysAlisPro program complex using spherical harmonics, implemented in the SCALE3 ABSPACK scaling algorithm (CrysAlisPro, version 1.171.36.32; Agilent Technologies, Santa Clara, CA, USA). Supplementary crystallographic data have been deposited at Cambridge Crystallographic Data Centre, CCDC Number 1962711. It can be obtained free of charge via www.ccdc.cam.ac.uk/data_request/cif.

### 2.4. Bioassays

#### 2.4.1. Phytotoxic Activity

Leaf segments of couch-grass (*Elytrigia repens*) of 2 cm length and leaf discs of perennial sowthistle (*Sonchus arvensis*) of 1 cm in diameter were punctured with a sharp needle in the center and were treated with solutions of isolated compounds in 5% EtOH at a concentration of 2 mg/mL as described previously [31]. Typically, for this bioassay samples of isolated compounds (400 μg) were dissolved in EtOH (10 μL) ultrasonically, and then, water (190 μL) was added and the samples were vortexed. At least 10 replicate leaf segments or discs were used for each treatment. The treated disks were incubated at 24 °C for 48 h, the phytotoxic activity was determined as diameter and length of necrotic lesions for sowthistle and couch-grass respectively. Metribuzin (Sigma-Aldrich, Schnelldorf, Germany) an herbicidal compound from the triazinone group was used for a positive treatment.

#### 2.4.2. Antimicrobial Activity

The antibacterial activity of the isolated compounds was tested against *Bacillus subtilis* (Gram-positive bacterium) and *Escherichia coli* (Gram-negative bacterium) obtained from The Russian Collection of Agricultural Microorganisms (Pushkin, Saint-Petersburg, Russia). Ampicillin (a wide spectrum antibiotic) (Sigma-Aldrich, Schnelldorf, Germany) was used as a positive control. The antifungal activity of *A. sonchi* metabolites was evaluated against a non-pathogenic strain of the yeast, *Candida tropicalis* provided by Professor O. Vinokhodov (Saint-Petersburg State Technical University, Saint-Petersburg, Russia). The bioassays were performed by the paper-disc agar diffusion technique according to the protocol previously reported [32]. The microorganisms were grown on potato-dextrose agar. Up to 100 μg of each metabolite was applied per disc (6 mm in diameter, Macherey-Nagel, Düren, Germany). The treated microbial cultures were incubated at 30°C for 24 h, and radius of growth inhibition zone was measured. The antimicrobial activity of tested compounds was expressed as minimal inhibition concentration (MIC, μg/disc).

#### 2.4.3. Cytotoxic Activity

Human histiocytic lymphoma (U937) and human myelogenous leukemia (K562) cell lines were obtained from the Russian Cell Culture Collection (Institute of Cytology of the Russian Academy of Science, Saint-Petersburg, Russia). Cell cultures were grown in RPMI 1640 medium (Biolot, Saint-Petersburg, Russia) supplemented with 10% fetal bovine serum HyClone (GE Healthcare, Marlborough, MA, USA), 2 mM GlutaMAX (Gibco, Thermo Fisher Scientific, Waltham, MA, USA) at 37 °C and 5% CO_2_. Cells were incubated for 48 h in 96-well plates with 200 μL medium containing test extracts (5–100 μg/mL) in each well. Then, cells were stained with YO-PRO^®^-1 and propidium iodide (Invitrogen, Thermo Fisher Scientific, Waltham, MA, USA), as described in [33]. The viability of cells in test and control wells was evaluated by flow cytometry. The analysis of data was conducted by means of the CytExpert program (Beckman Coulter, Fullerton, CA, USA). For statistical analysis and IC_50_ determination GraphPad Prism (GraphPad Software, Inc., San Diego, CA, USA) have been used. Etoposide (Sigma-Aldrich, Schnelldorf, Germany), a semisynthetic derivative of the plant metabolite, podophyllotoxin was used as a positive control.

#### 2.4.4. Insecticidal Activity

Contact insecticidal activity of *A. sonchi* metabolites was assessed against wheat aphid (*Schizaphis graminum*) at a concentration of 1 mg/mL in 5% EtOH. Filter paper discs (4 cm in diam.) were moistened with 250 μL of test solutions each receiving the final concentration 20 μg/cm^2^ of tested compounds. The moistened discs placed on both the bottom and the lid of Petri dishes. A 2-cm segment of wheat leaf was dipped into the test solution and placed into each Petri dish. Then, 20 aphids were introduced to the each of prepared Petri dishes [34]. Aphid mortality was measured 24 h after treatment, and the insecticidal efficacy of isolated compounds was calculated by Abbott’s formula [35]. Imidacloprid (Dr. Ehrenstorfer GmbH, Augsburg, Germany), an insecticidal compound from the group of neonicotinoids, and beauvericin (Sigma-Aldrich, Schnelldorf, Germany), a mycotoxin produced by some toxigenic *Fusarium* spp. and enthomopathogenic *Beauveria* spp., were used for a comparison.

#### 2.4.5. Zootoxicity Assay

The zootoxic activity of compounds **1**–**13** was evaluated on the infusoria, *Paramecium caudatum* as a model organism [36]. The isolated compounds were tested at concentrations of 1, 10, and 100 μg/mL in 5% EtOH. The assay was performed in a 24-well plate. A suspension of *P. caudatum* cells (900 μL) and 100 μL of stock solutions of **1**–**13** prepared in 50% EtOH were poured to each well. In the control treatment, 100 μL of 50% EtOH was added to 900 μL of the paramecia suspension. The *P. caudatum* culture was incubated at 24 ± 1 °C. Toxic effects of **1**–**13** on paramecia movement was observed after 3, 30, and 180 min post treatment. If 100% of the infusoria become immobile within the mentioned period of time, the compounds were estimated to be highly, medium, and low toxic, respectively.

#### 2.4.6. Esterase Inhibitory Activity

The enzymes, carboxylesterase (CE) and butyrylcholinesterase (BCE) from the Sigma catalogue (E3019 and B4186, respectively) were used. The isolated compounds **1**–**4**, **6**, **7**, **9**, **13** were dissolved in DMSO to adjust the concentration to 10 mg/mL and then diluted with 10 mM phosphate buffer saline (pH 7.4) containing 137 mM NaCl and 2.7 mM KCl (PBS, Biolot) to adjust the concentration to 1 mg/mL. The enzyme solutions in PBS, with the activity of 25–30 U, and the compounds solutions at a 9:1 volume ratio were introduced into the wells of a 96-well plate. Thus, the end concentration of the tested compounds in the wells was 100 μg/mL. The equivalent PBS volume was introduced into the control wells instead of the metabolite solutions. The test solutions were incubated with the enzymes for 1 h at 25 °C. The plates were then heated on a temperature-controlled platelet shaker at 37 °C for 5 min, and the activity of serine esterases was determined at the same temperature in the kinetic mode in the presence of excess substrate using conventional photometric methods with insignificant modifications [37,38]. To assess CE and BCE activity, p-nitrophenylacetate (p-NPA) and butyrylthiocholine were used as the respective substrates. Spontaneous substrate degradation was considered in the course of calculations. All bioassays were performed in triplicate. Esterase-inhibition activity was expressed as % of untreated control. 2-(2-Cresyl)-4H-1-3-2-benzodioxaphosphorin-2-oxide (CBDP) and tetraisopropyl pyrophosphoramide (iso-OMPA) (both from Sigma-Aldrich, Schnelldorf, Germany) were used as the positive control for inhibition of CE and BCE, respectively.

## 3. Results

### 3.1. Structure Elucidation

Thirteen compounds (**1**–**13**) were purified from the organic extract of solid culture of *A. sonchi* S-102 using various chromatographic techniques. The structures of two new natural compounds, chlorinated xanthone (**1**) and benzophenone derivative chloromoniliphenone (**2**), were elucidated by interpretation of spectroscopic data, including UV, NMR and MS. The analysis of MS-spectra of **1** and **2** showed the presence of the [37] Cl isotope supposing both to be monochlorinated compounds.

**Figure 2:**
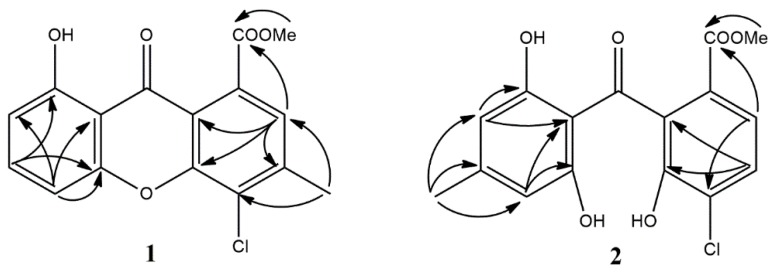
The selected HMBC correlations of compounds **1** and **2**.

**Figure 3:**
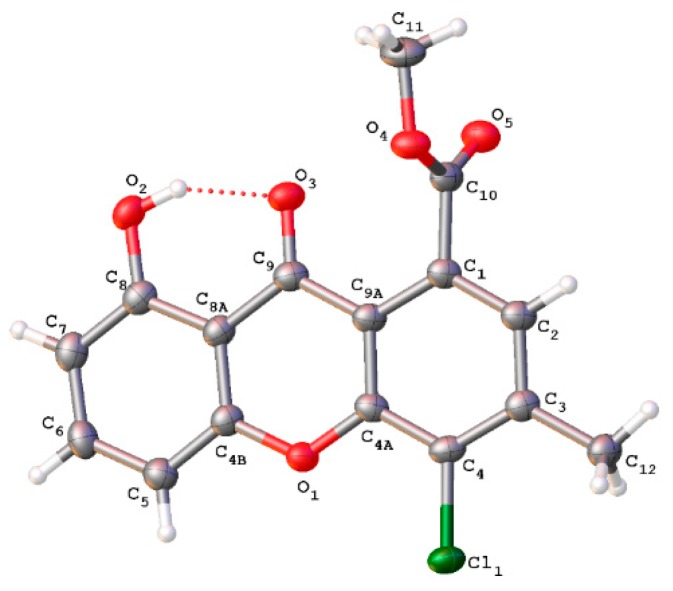
ORTEP view of methyl 8-dihydroxy-3-methyl-4-chloro-9-oxo-9H-xanthene-1-carboxylate (**1**), showing atomic labeling. Displacement ellipsoids are drawn at the 30% probability level.

Compound **1** was isolated as pale-yellow crystals. Molecular formula of **1** was determined to be C_16_H_11_ClO_5_ by HRESIMS (*m/z* 341.0172 [M + Na]^+^, calcd 341.0193). Its UV spectrum displayed absorption bands at λ_max_ 237, 255, 306, and 366 nm, similar to pinselin (**10**) [39] and other xanthones, suggesting that both compounds share the same core structure. The ^1^H NMR spectrum of **1** (Table 1) exhibited four aromatic protons H-2, H-5, H-6 and H-7 at *δ*_H_ 7.25 (1H, s), 6.88 (1H, d, *J* = 8 Hz), 7.66 (1H, t, *J* = 8 Hz), and 7.06 (1H, d, *J* = 8 Hz), respectively; one methoxy group at *δ*_H_ 4.04 (3H, s), one methyl group at *δ*_H_ 2.60 (3H, s), and a hydroxyl at *δ*_H_ 12.13 ppm (1H, s). Moreover, ^13^C NMR data (Table 1) along with HSQC and HMBC spectral analysis revealed the presence of 16 carbons, including one chelated carbonyl group (*δ*_C_ 180.5), one carboxyl group (*δ*_C_ 169.0), eight quaternary carbons (*δ*_C_ 161.7, 155.5, 151.9, 144.7, 131.1, 124.0, 116.7, 108.5), four tertiary carbons (*δ*_C_ 137.3, 124.4, 111.5, 107.2), a methoxy group (*δ*_C_ 53.3), and a methyl group (*δ*_C_ 22.6). The methyl group (*δ*_H_ 2.60) was located at C-3 from the HMBC correlation with C-2 (*δ*_C_ 124.4) and C-4 (*δ*_C_ 131.1) (Figure 2). In the HMBC spectrum of **1**, the aromatic proton H-2 (*δ*_H_ 7.25) exhibited correlations to the ester carbonyl group (*δ*_C_ 168.9), C-3 (*δ*_C_ 144.7), C-4a (*δ*_C_ 151.9), and C-9a (*δ*_C_ 116.7) (Figure 2). Three aromatic protons at *δ*_H_ 6.88, 7.66, and 7.06 were attributed to H-5, H-6, and H-7, respectively, based on their multiplicities and HMBC correlations (Figure 2). From these results, **1** was determined to be methyl 8-dihydroxy-3-methyl-4-chloro-9-oxo-9*H*-xanthene-1-carboxylate (Figure 1). The X-ray data confirmed this molecular structure (Figure 3).

Compound **2** was obtained as a yellowish oil. Molecular formula of **2** was determined to be C_16_H_13_ClO_6_ by HRESIMS (*m/z* 359.0280 [M + Na], calcd 359.0298). The UV spectrum showed absorption bands at 205, 247, 287, and 351 nm, which was similar to those of moniliphenone (**4**) [40]. The ^1^H and ^13^C NMR spectrum of **2** indicated the presence of a methyl (*δ*_H_ 2.26 s, *δ*_C_ 22.1); methoxy group (*δ*_H_ 3.76 s, *δ*_C_ 52.6); four tertiary aromatic carbon (overlapped *δ*_H_ 6.23 s, *δ*_C_ 109.3; *δ*_H_ 7.56 (1H, d, *J* = 8 Hz), *δ*_C_ 122.7; *δ*_H_ 7.43 (1H, d, *J* = 8 Hz), *δ*_C_ 128.9); one carbonyl group (*δ*_C_ 197.0); one carboxyl group (*δ*_C_ 166.0); and eight quaternary carbons (overlapped signals at *δ*_C_ 160.5, *δ*_C_ 148.9, 147.8, 132.3, 127.5, 125.2, 109.1). Comparison of the molecular formula and NMR data of **2** with those of co-isolated moniliphenone (**9**) revealed that **2** differs from **9** in the presence of a chlorine atom instead of an aromatic proton. Two aromatic protons resonating at *δ*_H_ 7.56 and 7.43 were assigned as H-2 and H-3, respectively, on the basis of their multiplicities, coupling constants and HMBC correlations (Table 2, Figure 2). These data presumed **2** to be benzophenone derivative 5-chloromoniliphenone (Figure 1).

Eleven known compounds were identified comparing original and literature spectroscopy data: 4-chloropinselin (**3**) [41], methyl 3,8-dihydroxy-6-methyl-4-chloro-9-oxo-9*H*-xanthene-1-carboxylate (**4**) [42], pinselin (**5**) [39], methyl 3,8-dihydroxy-6-methyl-9-oxo-9*H*-xanthene-1-carboxylate (**6**) [43], methyl 8-hydroxy-6-methyl-9-oxo-9*H*-xanthene-1-carboxylate (**7**) [40], chloromonilicin (**8**) [27], moniliphenone (**9**) [40], chloromonilinic acids B (**10)**, C (**12**), and D (**11**) [44,45], and α-, β-diversolonic esters (**13**) [46] (Figure 1). Among them, compounds **4**–**7**, **9**, **11**, and **13** were first reported for *A. sonchi*.

Methyl 3,8-dihydroxy-6-methyl-9-oxo-9*H*-xanthene-1-carboxylate (**6**) was previously isolated from *Aspergillus wentii* [41] and *Microsphaeropsis* sp. [47]. However, the published spectroscopic data were insufficient and included ^1^H-NMR and IR spectra only [43]. Therefore, the relevant data are given in the Materials and Methods section and support the structure of **6**.

**Figure 4:**
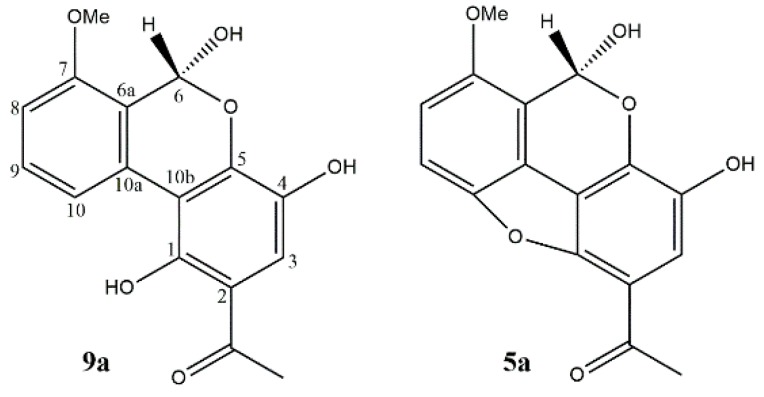
Structures of alternethanoxin A (**9a**) and alternethanoxin B (**5a**).

All the isolated compounds from *A. sonchi* xanthones, chromones, and benzophenones are structurally and biosynthetically related [42]. Moreover, it was noted that UV- and MS-spectra of **5** and **9** are identical to alternethanoxins A and B (**5a** and **9a**), respectively [24,39,40]. This led us to revise structures of alternethanoxins A and B (Figure 4).

The comparison of newly isolated compounds **9** and **5** with stored samples of alternethanoxins A (**9a**) and B (**5a**) by UPLC-PDA, HPLC-ESIMS, and TLC confirmed their identity. The UV spectra of previously reported alternethanoxin A (λ_max_ 381, 299, 241 nm) and alternethanoxin B (λ_max_ 381, 294, 262, 237 nm) [24] are typical for benzophenones and xanthones, respectively [47,48,49,50]. Detailed checking the NMR assignments supported a revision of the structure **9a** to moniliphenone (**9**) and **5a** to pinselin (**5**) (Table 1 and Table 2). The observed overlapping signals at *δ*_H_ 6.21 (2H, s) were assigned as H-6 and H-3 in previously reported structure **9a [24]**. These protons have different chemical environments in alternethanoxin A (**9a**), but in **9,** these overlapping signals at *δ*_H_ 6.21 were reassigned to aromatic protons H-10 and H-12 that are located in symmetrical fragment of benzophenone molecule.

### 3.2. Biological Activity

The isolated compounds **1**–**13** were subjected to a number of bioassays and showed various types of biological activity.

Compounds **2**, **3**, **9**, and **10** showed relatively low nonspecific phytotoxic activity at a concentration of 2 mg/mL on punctured leaf segments of both *Sonchus arvensis* and *Elytrigia repens*, expressed as necrotic lesions about 1–2.5 mm in diameter or length, respectively. Leaves of *E. repens* were more sensitive to chromone derivatives **10**–**12** than leaves of the fungus host plant, *S. arvensis*. Among xanthone derivatives only **3** was toxic to both plants, xanthones **1**, **4**–**7** showed no activity (Table 3). Metribuzin was non-toxic to *E. repens* leaves, while its activity to *S. arvensis* (lesions about 2.5 mm in diam.) was comparable to compound **2**.

Isolated compounds did not affect the growth of seedling roots of both wheat and radish at concentrations up to 100 μg/mL. Among the 11 compounds tested (**1**–**6**, **8**–**11**, and **13**), only chloromonilicin (**8**) inhibited the growth of seedling roots of lettuce at a concentration of 100 μg/mL, decreasing their length on about 30% compared with the control (data not shown).

Compounds **4**, **8**, and **9** showed antimicrobial activity against *Bacillus subtilis*, *Escherichia coli*, and *Candida tropicalis* used in this study (Table 3). Chloromonilicin (**8**) was found to possess the highest antimicrobial activity with MIC values of < 0.5 μg/disc against *B. subtilis* and *E. coli*, 1.0 μg/disc against *C. tropicalis*. 5-chloromoniliphenone (**2**) and α-, β-diversolonic esters (**13**) displayed antimicrobial activity against both *B. subtilis* and *C. tropicalis* at 10–50 μg/disc. Compounds **10**–**12** inhibited the growth of *B. subtilis* at MIC 100 *μ*g/disc (Table 3). MIC of ampicillin against *Bacillus subtilis* and *Escherichia coli* was < 0.5 μg/disc that was similar to activity of **8** against these bacteria.

Most compounds from the *A. sonchi* solid culture possessed contact insecticidal activity against wheat aphid (*Schizaphis graminum*). Insecticidal efficacy of methyl 3,8-dihydroxy-6-methyl-4-chloro-9-oxo-9*H*-xanthene-1-carboxylate (**4**) and chloromonilic acid B (**10**) was above 70% when tested at a concentration of 1 mg/mL. Compounds **1**, **3**, **5**, **6**, **9**, and **12** showed relatively moderate insecticidal efficacy in the range of 30–70% (Table 3). In the same bioassay, imidacloprid killed 100% of the aphids at a concentration of 50 µg/mL, while beauvericin was similarly active at the 10 times higher concentration of 500 µg/mL.

Some *A. sonchi* metabolites were tested for anti-esterase activity. Compounds **2**, **3**, and **6** demonstrated more than 50% inhibition of CE, while BCE activity was not considerably inhibited at a concentration of 100 μg/mL. Methyl-3,8-dihydroxy-6-methyl-9-oxo-9*H*-xanthene-1-carboxylate (**6**) was shown to be the most potent as a selective CE inhibitor (Table 4). In the positive control, CBDP fully inhibited CE activity at a concentration of 1 μg/mL, while BCE was fully inhibited by iso-OMPA at a concentration of 20 μg/mL.

Metabolites of *A. sonchi* were moderate (**3**–**7**), low (**2**, **11**) or non-toxic to the infusoria, *Paramecium caudatum*, at the highest concentration tested of 100 μg/mL. Despite that xanthone derivatives **3**–**7** killed the infusoria after 30 min of exposure, their effect seems mechanical due to the fast crystallization in 5%-EtOH. At a concentration ≤10 μg/mL, the xanthones did not show toxicity to *P. caudatum*. Compounds **2** and **11** caused gradual deceleration of the infusoria motion after 3 h of exposure (data not shown).

Isolated compounds, except for **8** (due to insufficient material being available), were tested for cytotoxic activity against human histiocytic lymphoma (U937) and human myelogenous leukemia (K562) cell lines (Table 5). The compounds **4** and **6** displayed the most cytotoxic activity against U937 cells (IC_50_ 72 and 90 µM respectively); however, with much lower levels, as etoposide was used as positive control. Compounds **4**, **6**, **12**, and **13** demonstrated low level of cytotoxicity against K562 cell line with IC_50_ >100 µM (Table 4). When **1**–**7**, **9**, and **13** were additionally tested using cell lines A549 and U251, only methyl 3,8-dihydroxy-6-methyl-4-chloro-9-oxo-9*H*-xanthene-1-carboxylate (**4**) was cytotoxic with IC_50_ about 65 µg/mL (>100 μM) (data not shown).

## 4. Discussion

Xanthones and their biosynthetic relatives (anthraquinones, benzophenones, chromones) are common fungal secondary metabolites [51]. Compounds 3–13 have been reported to be produced by different fungi (Table 6). Nevertheless, the chlorinated xanthones with simple skeleton were mainly isolated from lichens [52]. Examples of such fungal chlorinated xanthones are 4-chloropinselin from *Monilinia fructicola* and *Bipolaris sorokiniana* [40,42], chloroisosulochrin dehydrate from *P. theae* [49], 3,6,8-trihydroxy-5-chlorin-1-methylxanthone from *Stachybotrys* sp. [53], 4-chlorofischexanthone from *Alternaria* sp. [54].

Biosynthetic and biodegradation route of chloromonilicin (**8**) in *M. fructicola* investigated by Kachi and Sassa [40], Horiguchi et al. [41], Sassa et al. [44], Sassa [55]. The biosynthetic pathway of **9** to **8**, via **5** and **3**, involves hydroxylation and chlorination of the phenolic nucleus, ring closure of the benzophenone structure to a xanthone type [41]. Recently, Han et al. isolated new related compounds from phytopathogenic fungus *B. sorokiniana* and characterized the gene cluster encoding halogenated polyketides [42]. A similar range of halogenated metabolites and their pre-cursors isolated from *A. sonchi*, *M. fructicola*, and *B. sorokiniana* indicates that their biosynthesis proceeds in a similar way. Interestingly, compound **1** differs from other described xanthones by location of methyl group at C-3 instead of C-6. An example of such xanthone derivatives is 8-hydroxy-3-methylxanthone-1-carboxylate from *Apiospora montagnei* [56]. Therefore, **1** was suggested to be derived from chrysophanol [42]. The production of 5-chloromoniliphenone was predicted in *B. sorokiniana* as a biosynthetic intermediate of 4-chloropinselin and chloromonilicin [42]. This compound was isolated and fully characterized as a metabolite of *A. sonchi* and, therefore, reported for the first time as a natural product.

The metabolites isolated from the solid culture of *A. sonchi* on pearl barley did not demonstrate high phytotoxicity (lesions up to 2.5 mm in length/diameter at a concentration of 2 mg/mL) and cytotoxicity (IC_50_ > 25 µg/mL). They did not possess acute toxicity to *Paramecium caudatum*, a model single-cell organism for toxicological studies [36]. However, some of them were shown to have antimicrobial activity with MIC 0.5–5 µg/disc (**4**, **8**) and relatively moderate insecticidal properties (**4**, **10**) at 1 mg/mL.

It seems the residual substrate remained after sporulation of *A. sonchi*, which contains the fungal metabolites and is not hazardous for environment. Our preliminary observations indicated chloromonilicin (**8**), the main antimicrobial component of the extract is quite unstable (data not shown). Therefore, the waste material after production of a mycoherbicide based on *A. sonchi* conidia could be destroyed without special techniques. Moreover, it can be re-used for the production of useful by-products (e.g., **4**, **6**, **8** or **10**). The major fungal metabolite, 4-chloropinselin (**3**), could be a precursor for semi-synthetic biologically active chlorinated xanthones, as it has been shown for α-mangostin from *Garcinia mangostana* [57].

Generally, the natural xanthones described in literature showed selective cytotoxicity ranging from moderate to low activity against certain cancer cell lines. Pinselin (**5**) displayed immunosuppressive activity against the Con A-induced proliferation with IC_50_ value of 26.75 μg/mL, and lipopolysaccharide-induced with 30.68 μg/mL. [58] Moreover, it showed cytotoxicity against five human tumor cell lines—NB4, A549, SHSY5Y, PC3, MCF7—with IC_50_ value of 6.1, 5.2, 8.7, >10, and >10 μM, respectively [59], but was inactive against KB, NCI-H137, and noncancerous Vero cell lines [60]. Methyl 8-hydroxy-6-methyl-9-oxo-9H-xanthene-1-carboxylate (**7**) was non-toxic for KB, MCF-7, and NCI-H137 cell lines [56]. Moniliphenone (**9**) displayed cytotoxicity against human tumor cell lines MCF-7, H460, and SF268 with IC_50_ values of > 25 μM [61]. The previously reported alternethanoxin B (**5a**), which in the present paper was identified as pinselin (**5**), also demonstrated cytotoxic activity for OE21, Hs683, and A549 cell lines but with the higher IC_50_ than reported by Yang et al. [59] (64, 71, and 80 μM, respectively) [62]. The broad spectrum of fungal xanthones and related compounds produced by *A. sonchi* provides the useful information about structure-activity relationships of these compounds. Compounds **4** and **6** displayed cytotoxic activity against U937 and K562 cell lines, the common feature of these compounds is hydroxyl group attached to C-3. 2,6-dihydroxy-3-methyl-9-oxoxanthene-8-carboxylic acid methyl ester isolated from the marine fungus *Phomopsis* sp. by Yang et al. showed cytotoxicity against HepG2 and HEp-2 cells (IC_50_ = 30 and 26.7 μM, respectively) [59]. At the same time, closely related conioxanthone A from *Penicillium citrinum* did not demonstrate toxicity to KB, MCF-7, and NCI-H137 cell lines [56]. In Table 6, we summarized yields and properties of **1**–**13**.

Masi et al. [45] showed phytotoxic activity of chloromonilinic acids B (**10**), C (**12**), and D (**11**) on buffelgrass seedling. Phytotoxic activity of some synthetic benzophenones was described in literature as well [63]; however, such compounds have not been isolated from phytopathogenic fungi as phytotoxins. The ecological role of the secondary metabolites in the life cycle of *A. sonchi* remains unclear, but it cannot be ruled out that isolated phytotoxic compounds play a role in pathogenesis of the fungus on *Sonchus arvensis* leaves.

Finally, it should be noted that spores of many mycoherbicidal fungi, especially *Alternaria* spp., are produced by solid-state fermentation (SSF). These micromycetes are well-known as a source of both valuable metabolites and mycotoxins [64,65]. Moreover, SSF is used widely for production of other biopesticides: biofungicides and bioinsecticides [66,67,68]. At the same time, biocontrol fungi such as *Beauveria* and *Trichoderma* spp. are producers of enormous amounts of useful metabolites [69,70]. Therefore, an approach of simultaneous production of spores and useful metabolites shown in this study can be applied for many biocontrol microorganisms in the future.

## 5. Conclusions

Two new natural compounds, namely methyl 8-dihydroxy-3-methyl-4-chloro-9-oxo-9*H*-xanthene-1-carboxylate (**1**) and 5-chloromoniliphenone (**2**), were isolated from pearl barley culture of *A. sonchi* together with eleven known biosynthetically related fungal metabolites. These compounds were assayed for various types of biological activity and found to be low phytotoxic and cytotoxic. Some compounds showed interesting antimicrobial, insecticidal, and esterase-inhibition activity. It may indicate that waste solid substrate for production of *A. sonchi* spores is relatively ecologically safe and can be re-utilized for isolation of a number of natural products such as 4-chlorpinselin as a scaffold for semisynthetic xanthones. The results of this study can encourage both the use of biopesticides and re-utilization of waste culture substrates for isolation of valuable natural products.

## Figures and Tables

**Table 1:** ^1^H and ^13^C NMR data for xanthones **1** and **5** (at 400 and 100 MHz respectively, in CDCl_3_, δ in ppm).

Position (in 1 and 5)	Literature Assignment (5a) [24]	1		5	
		*δ*_H_ (*J* in Hz)	*δ*_C_	*δ*_H_ (*J* in Hz)	*δ*_C_
1	1		124.0	-	148.8
2	5	7.25 1H, s	124.4	-	155.7
3	8		144.7	7.40 1H, d (9.0)	125.4
4	9		131.1	7.45 1H, d (9.0)	121.7
4a	6a		151.9	-	150.5
4b	4		155.7	-	161.2
5	6	6.88 1H, d (8.0)	111.5	6.64 1H, s	111.3
6	10	7.66 1H, t (8.0)	137.3	-	151.9
7	3	7.06 1H, d (8.0)	107.2	6.75 1H, s	107.2
8	10a		161.7	-	155.7
8a	2		108.5	-	114.7
9	MeCO		180.5	-	180.3
9a	10b		116.7	-	118.5
10	7		169.0	-	168.9
11	OMe	4.04 3H, s	53.2	4.03 3H, s	53.1
12	MeCO	2.46 3H, s	20.9	2.45 3H, s	22.6
8-OH		12.10 s		12.23 s	

**Table 2:** ^1^H and ^13^C NMR data for benzophenones **2** and **9** (at 400 and 100 MHz respectively, in CDCl_3_, δ in ppm).

Position (in 2 and 9)	Literature Assignment (9a) [24]	2		9	
		*δ*_H_ (*J* in Hz)	*δ*_C_	*δ*_H_ (*J* in Hz)	*δ*_C_
1	10a		132.3		130.3
2	8		127.5		128.8
3	9	7.56 1H, d (8.5)	122.7	7.48 1H, d (7.5)	122.1
4	10	7.43 1H, d (8.5)	128.9	7.36 1H, t (7.5)	130.9
5	2		125.2	7.15 1H, d (7.5)	121.4
6	6a		147.8		153.6
7	COMe		197.0		199.0
8	10b		109.1		107.5
9	5		160.5		160.2
10	3	6.23 2H, s	109.3	6.24 2H, s	109.4
11	1		148.9		148.8
12	6	6.23 2H, s	109.3	6.24 2H, s	109.4
13	4		160.5		160.2
14	7		166.0		167.3
15	OMe	3.76 3H, s	52.6	3.65 3H, s	52.5
16	COMe	2.26 3H, s	22.1	2.25 3H, s	22.1

**Table 3:** Biological activity of isolated compounds **1**–**13**.

Compound	Phytotoxic		Antimicrobial ^3^			Insecticidal ^4^
	*Sonchus arvensis* ^1^	*Elytrigia repens* ^2^	*Bacillus subtilis*	*Escherichia coli*	*Candida tropicalis*	Wheat Aphid
**1**	0	0	NA	NA	NA	43.8 ± 12.1
**2**	2.4 ± 0.2	1.1 ± 0.3	10	NA	50	nt
**3**	1.5 ± 0.3	1.0 ± 0	NA	NA	NA	58.8 ± 17.8
**4**	0	0	<5	100	<5	80.0 ± 8.5
**5**	0	0.5 ± 0	NA	NA	NA	60.0 ± 4.8
**6**	0	0	NA	NA	NA	41.3 ± 8.7
**7**	0	0	NA	NA	NA	11.3 ± 6.5
**8**	0.9 ± 0.2	0	<0.5	<0.5	1	nt
**9**	1.7 ± 0.2	1.2 ± 0.4	20	20	100	42.5 ± 23.6
**10**	1.6 ± 0.5	2.0 ± 0.4	100	NA	NA	73.8 ± 9.1
**11**	0.7 ± 0.2	2.0 ± 0	50	NA	NA	NA
**12**	0.6 ± 0.2	1.7 ± 0.3	100	NA	NA	41.3 ± 12.6
**13**	0	0	50	NA	20	14.5 ± 9.4

^1^ diameter of necrotic lesion, mm; ^2^ length of necrotic lesion, mm; ^3^ minimal inhibitory concentration (MIC), μg/disc; ^4^ insecticidal efficacy, %. NA—no activity, nt—not tested.

**Table 4:** Esterase inhibition activity of compounds **1**–**7**, **9**, and **13**.

Compound	Anti-CE Activity, % ^1^	Anti-BCE Activity, % ^1^	CE/BCE Activity
**1**	61.3	89.3	0.69
**2**	14.1	83.8	0.17
**3**	28.7	80.6	0.36
**4**	65.7	91.5	0.72
**6**	2.9	62.6	0.05
**7**	55.4	80.6	0.69
**9**	68.6	55.1	1.24
**13**	91.3	96.7	0.94

^1^ % of control, CE—carboxylesterase, BCE—butyrylcholinesterase

**Table 5:** Cytotoxic activity of isolated compounds **1**–**13**.

Compound	IC_50_, µM	
	U937	K562
**1**	NA	NA
**2**	>100	>100
**3**	>100	>100
**4**	72	>100
**5**	NA	>100
**6**	90	>100
**7**	NA	NA
**8**	nt	nt
**9**	NA	NA
**10**	>100	NA
**11**	NA	NA
**12**	>100	>100
**13**	>100	>100
Etoposide	0.85	83

NA—no activity, nt—not tested.

**Table 6:** The yield and biological activity of *A. sonchi* metabolites.

Compound	Yield from Solid Culture of *A. sonchi*, mg/kg	Known Producers/Yield	Biological Activity
methyl 8-dihydroxy-3-methyl-4-chloro-9-oxo-9H-xanthene-1-carboxylate (**1**)	6	– *	Weak insecticidal *
5-chloromoniliphenone (**2**)	7	– *	Phytotoxic *, cytotoxic *, antiesterase *
4-chloropinselin (**3**)	1879	*Monilinia fructicola* [40]/15 mg/L; *Bipolaris sorokiniana* [42]/2 mg/kg;	Insecticidal *
methyl 3,8-dihydroxy-6-methyl-4-chloro-9-oxo-9H-xanthene-1-carboxylate (**4**)	29	*B. sorokiniana* [42]/1 mg/kg;	Antimicrobial [42], insecticidal *, cytotoxic *
pinselin (**5**)	223	*Penicillium amarum* [71]/ND *; *Aspergillus sydowii* [58]/4.4 mg/kg; *Phomopsis* sp. [59]/<1 mg/L; *Xylaria* sp. [72]/ND; *P. citrinum* [60]/<1 mg/L; *Engyodontium album* [73]/3.3 mg/kg; *Scopulariopsis* sp. [74]/1.8 mg/kg; *Cassia occidentalis* [75]/ND;	Immunosuppressive [58], cytotoxic [59], insecticidal *
methyl 3,8-dihydroxy-6-methyl-9-oxo-9H-xanthene-1-carboxylate (**6**)	56	*Microsphaeropsis* sp. [47]/<1 mg/L; *Scopulariopsis* sp. [74]/2.5 mg/kg; *Aspergillus iizukae* [76]/<1 mg/L;	antiviral [76];antiesterase *
methyl 8-hydroxy-6-methyl-9-oxo-9H-xanthene-1-carboxylate (**7**)	46	*M. fructicola* [40]/ND; *A. sydowii* [58]/2.3 mg/kg; *P. citrinum* [60]/<1 mg/L;	immunosuppressive [58]
chloromonilicin (**8**)	11	*M. fructicola* [40]/74 mg/L; *A. sonchi* [27]/ND; *Cochliobolus australiensis* [45]/2.23 mg/L;	antimicrobial [40], antifungal [27]
moniliphenone (**9**)	154	*M. fructicola* [40]/87.4 mg/L; *Hypocreales* MSX 17022 [61]/112 mg/kg; *A. sydowii* [58]/6.3 mg/kg; *P. citrinum* [60]/<1 mg/L; *Leptosphaeria* sp. [77]/26 mg/kg;	cytotoxic [61]
chloromonilinic acid B (**10)**	10	*M. fructicola* [44]/8 mg/L; *B. sorokiniana* [42]/<1 mg/L; *C. australiensis* [45]/16.8 mg/L;	phytotoxic [45], insecticidal *
chloromonilinic acid C (**12**)	7	*C. australiensis* [45]/3.0 mg/L;	phytotoxic [45], cytotoxic *
chloromonilinic acid D (**11**)	3	*C. australiensis* [45]/6.2 mg/L;	phytotoxic [45]
α- and β-diversolonic esters (**13**)	12	*P. diversum* [46]/2.4 mg/L; *P. citrinum* [60]/<1 mg/L	cytotoxic [61]

*—this study.

## Supplementary Materials

The following are available online at https://www.mdpi.com/2218-273X/10/1/81/s1, Figure S1: ^1^H NMR spectrum of compound **1**, Figure S1: ^1^H NMR spectrum of compound **1**, Figure S1: ^1^H NMR spectrum of compound **1**, Figure S2: ^13^C NMR spectrum of compound **1**, Figure S3: HSQC spectrum of compound **1**, Figure S4: HMBC spectrum of compound **1**, Figure S5: ESIMS of compound **1**, Figure S6: UV spectrum of compound **1**, Figure S7: ^1^H NMR spectrum of compound **2**, Figure S8: ^13^C NMR spectrum of compound **2**, Figure S9: HSQC spectrum of compound **2**, Figure S10: HMBC spectrum of compound **2**, Figure S11: HR ESIMS spectrum of compound **2**, Figure S12: UV spectrum of compound **2**, Figure S13: ^1^H NMR spectrum of compound **5**, Figure S14: ^13^C NMR spectrum of compound **5**, Figure S15: ^1^H NMR spectrum of compound **9**, Figure S16: ^13^C NMR spectrum of compound **9**, Table S1: X-ray data of compound **1**, are available free of charge on the Internet.
