# Supplementary material for: Isolation and Bioactivity of Secondary Metabolites from Solid Culture of the Fungus, Alternaria sonchi
Source: Biomolecules. 2020 Jan 4;10(1):81. doi: 10.3390/biom10010081 (PMC7022477; doi:10.3390/biom10010081)
Supplement: Supplementary file 1 [file biomolecules-10-00081-s001.pdf]

Supporting information

Isolation and Bioactivity of Chlorinated Xanthones and Chromones from Solid Culture of the Fungus, *Alternaria sonchi*, a Potential Bioherbicide for Control of Sowthistles

Anna A. Dalinova[1], Leonid S. Chisty[2], Dmitry M. Kochura[2], Varvara V. Garnyuk[2], Maria O. Petrova[1], Darya S. Prokophyeva[2], Anton N. Yurchenko[3], Vsevolod R. Dubovik[1], Alexander Y. Ivanov[4], Sergey N. Smirnov[4], Andrey A. Zolotarev[4] and Alexander O. Berestetskiy[1]

[1] All-Russian Institute of Plant Protection, Russian Academy of Agricultural Sciences, Pushkin, Saint-Petersburg 196608, Russian Federation

[2] Research Institute of Hygiene, Occupational Pathology and Human Ecology, Federal Medical Biological Agency, p/o Kuz'molovsky, Saint-Petersburg 188663, Russian Federation

[3] G.B. Elyakov Pacific Institute of Bioorganic Chemistry, Far Eastern Branch of Russian Academy of Sciences, Vladivostok 690022, Russian Federation

[4] St. Petersburg State University, Universitetsky Av. 26, St. Petersburg, 198504, Russian Federation

Figure S1 – ^{1}H NMR spectrum of compound **1**

Figure S2 – ^{13}C NMR spectrum of compound **1**

Figure S3 – HSQC spectrum of compound **1**

Figure S4 – HMBC spectrum of compound **1**

Figure S5 – ESIMS of compound **1** recorded in positive ion mode

Figure S6 – UV spectrum of compound **1**

Figure S7 – ^{1}H NMR spectrum of compound **2**

Figure S8 – ^{13}C NMR spectrum of compound **2**

Figure S9 – HSQC spectrum of compound **2**

Figure S10 – HMBC spectrum of compound **2**

Figure S11 – HR ESIMS of compound **2** recorded in positive ion mode

Figure S12 – UV spectrum of compound **2**

Figure S13 – ^{1}H NMR spectrum of compound **5**

Figure S14 – ^{13}C NMR spectrum of compound **5**

Figure S15 – ^{1}H NMR spectrum of compound **9**

Figure S16 – ^{13}C NMR spectrum of compound **9**

Table S1 – X-ray data of compound **1**

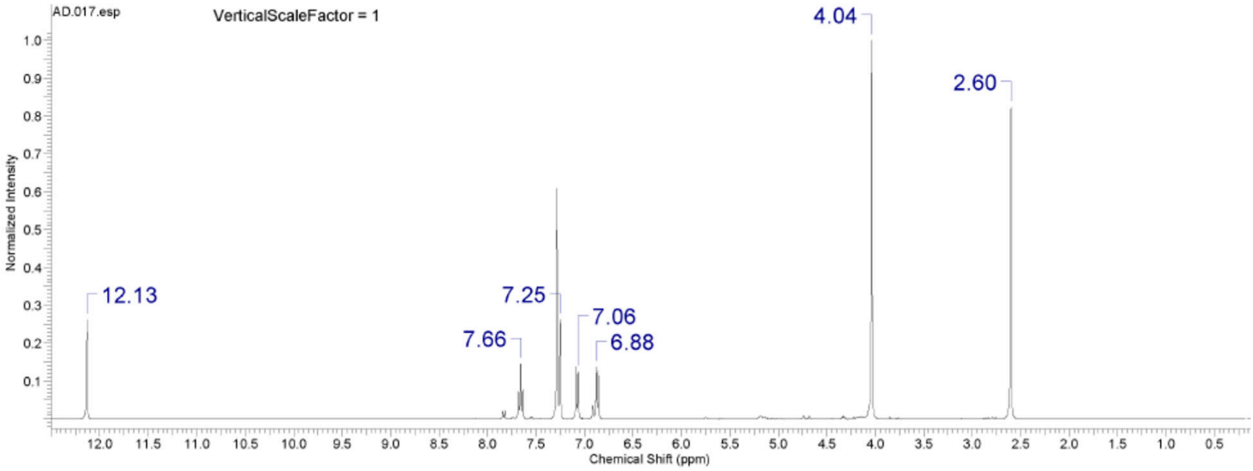

Figure S1 – ^{1}H NMR spectrum of compound **1**

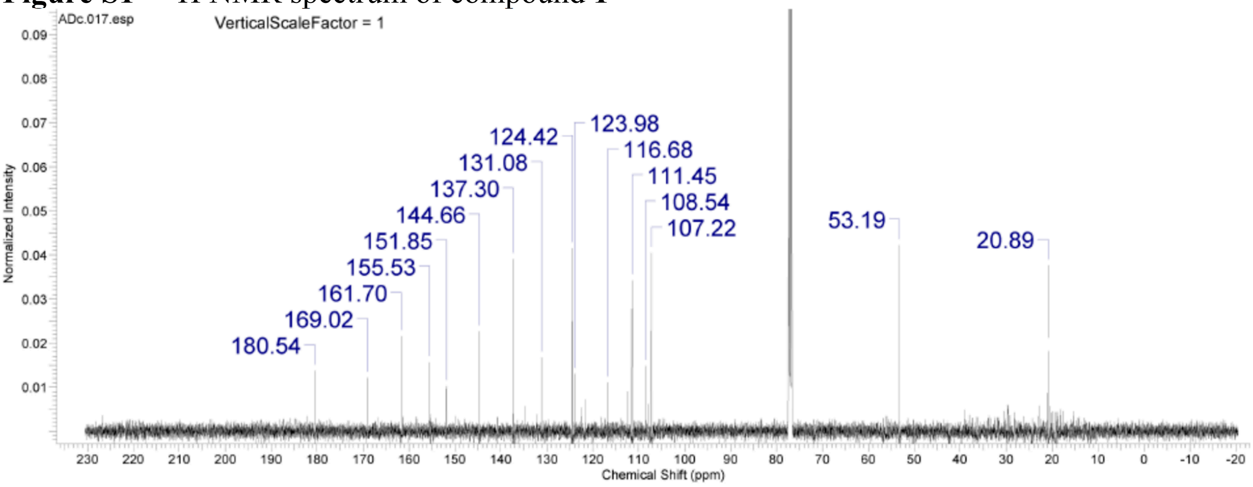

Figure S2 – ^{13}C NMR spectrum of compound **1**

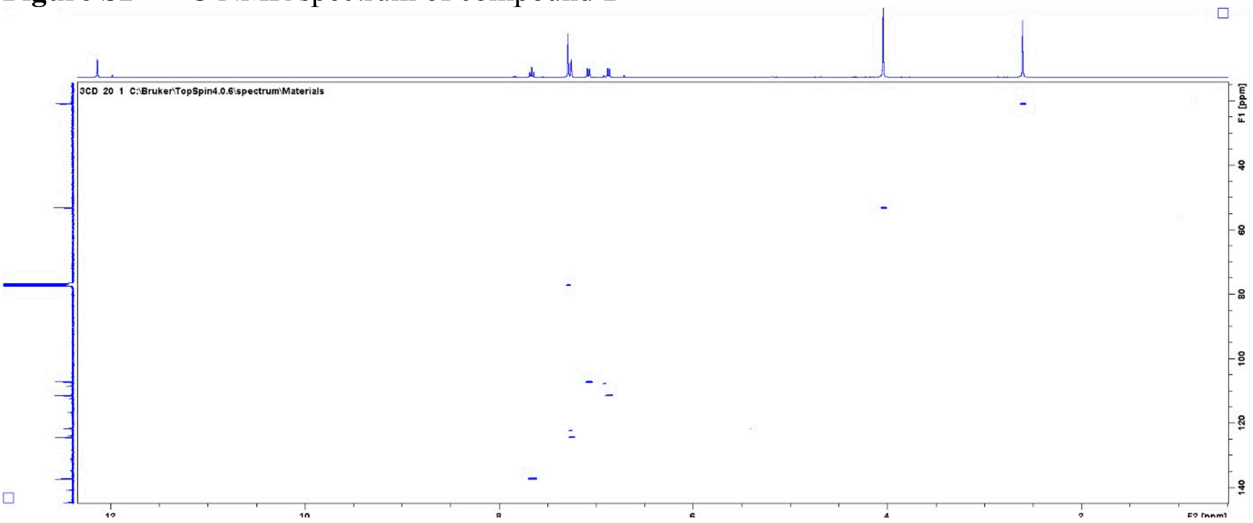

Figure S3 – HSQC spectrum of compound **1**

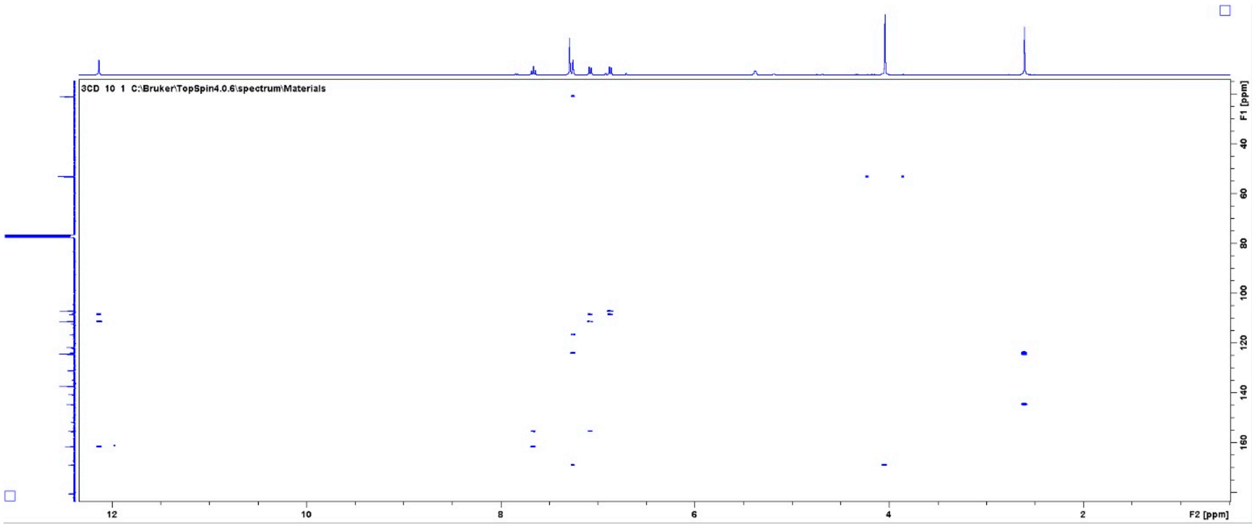

Figure S4 – HMBC spectrum of compound **1**

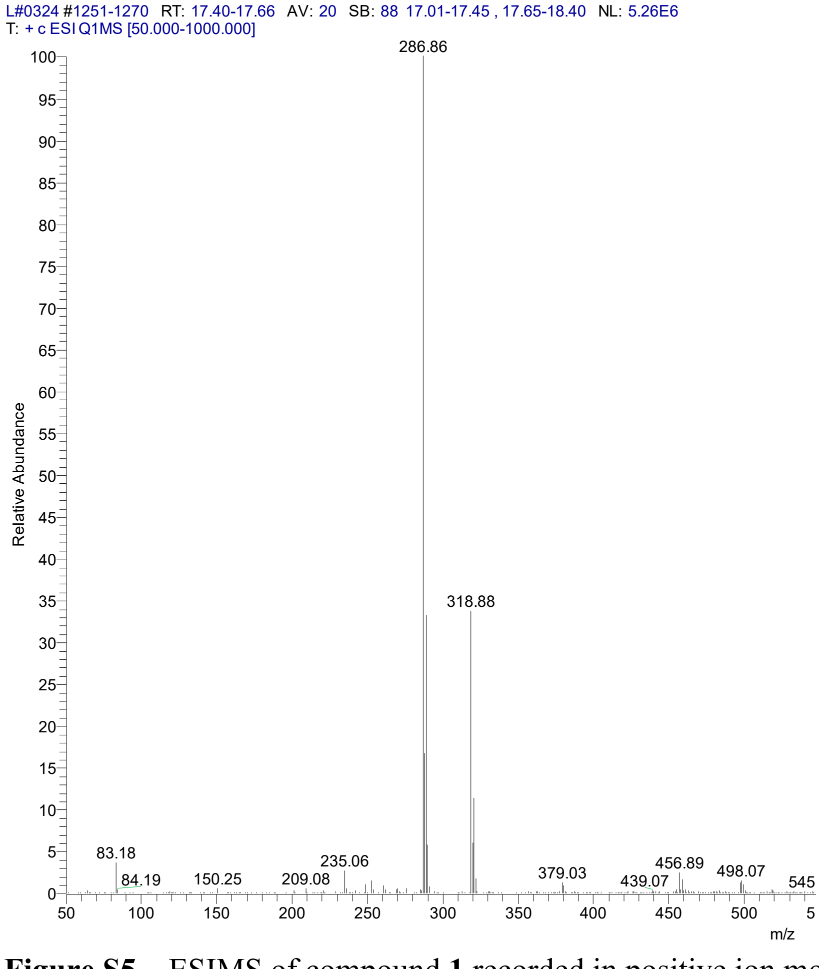

Figure S5 – ESIMS of compound **1** recorded in positive ion mode

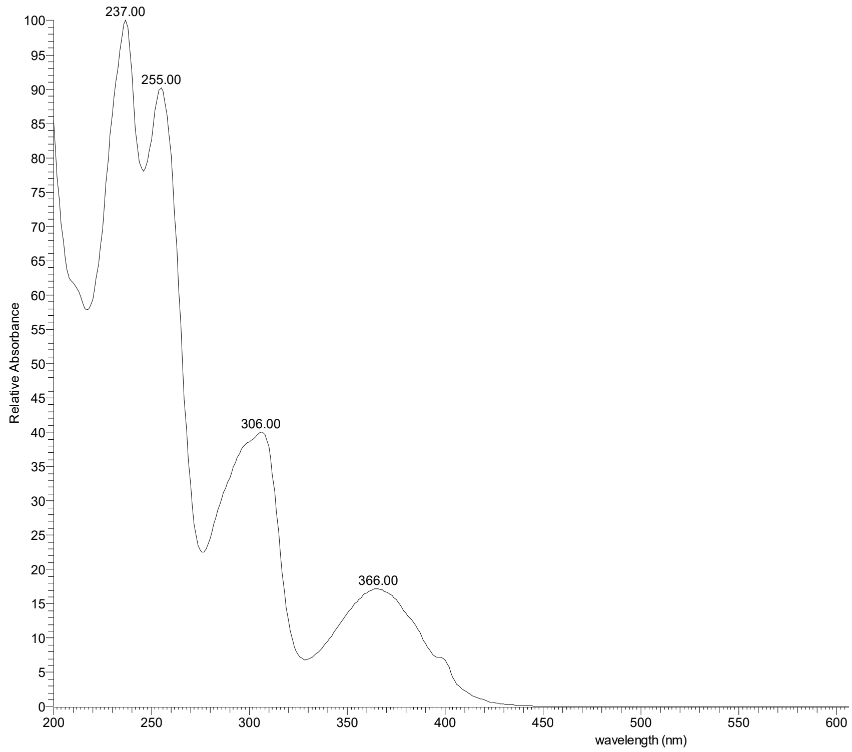

Figure S6 – UV spectrum of compound **1**

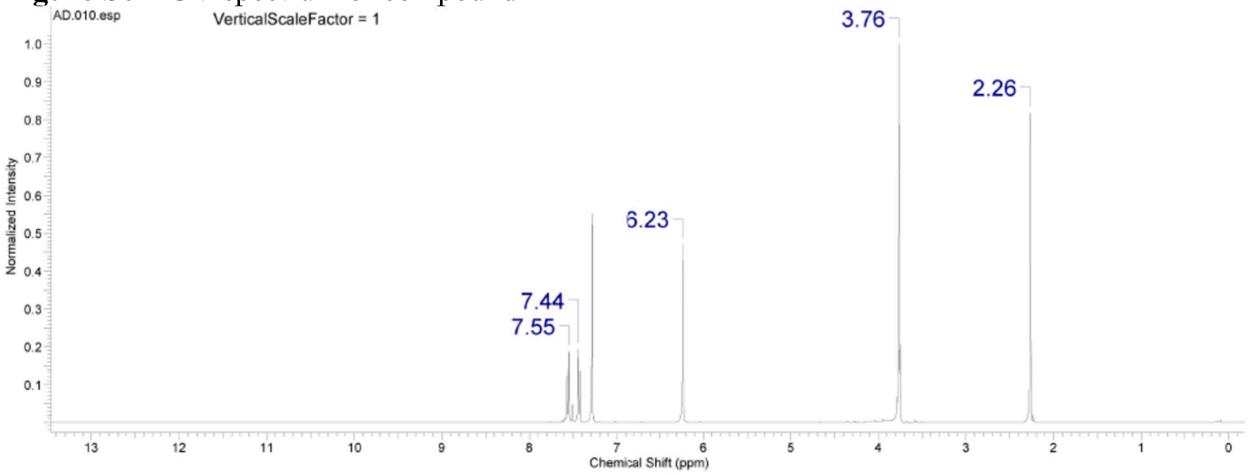

Figure S7 – ^{1}H NMR spectrum of compound **2**

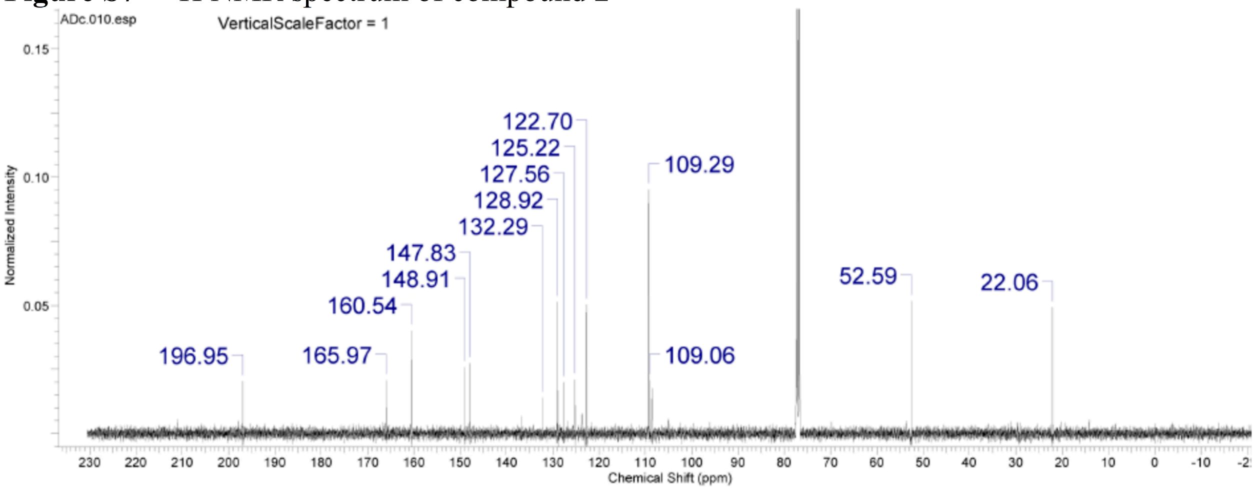

Figure S8 – ^{13}C NMR spectrum of compound **2**

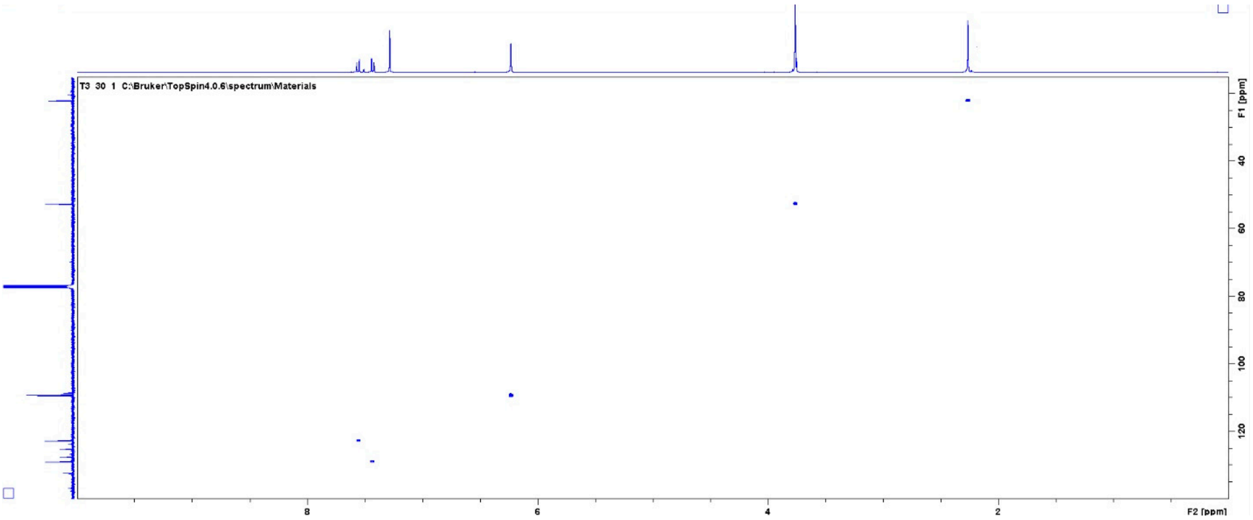

Figure S9 – HSQC spectrum of compound **2**

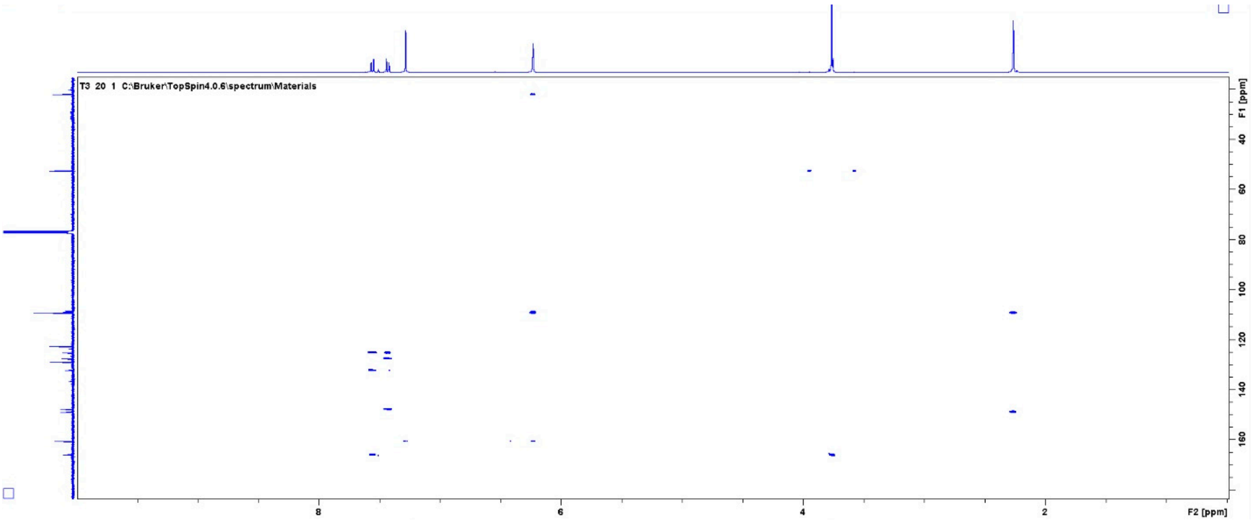

Figure S10 – HMBC spectrum of compound **2**

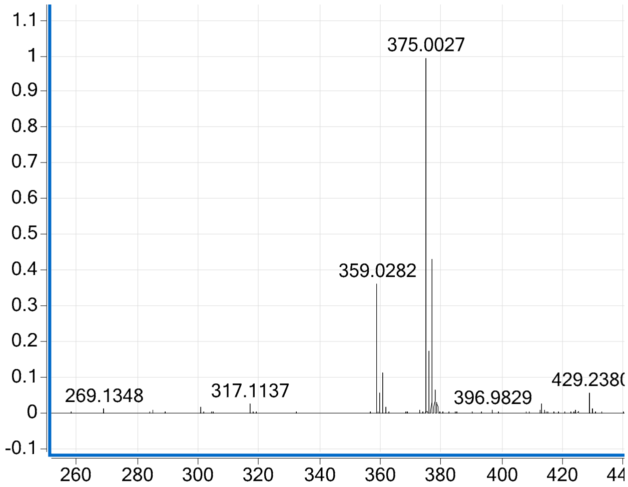

Figure S11 – HR ESIMS of compound **2** recorded in positive ion mode

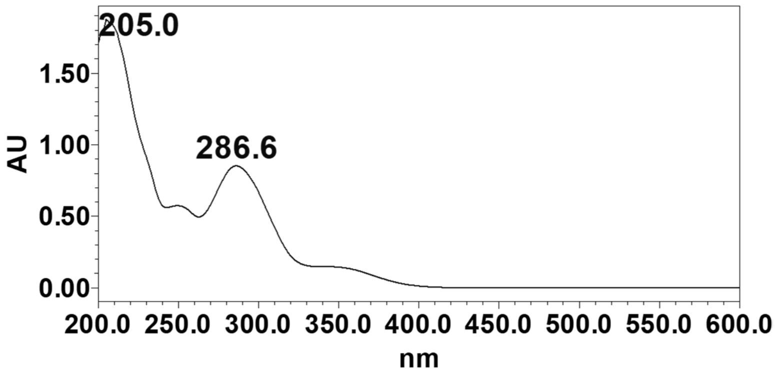

Figure S12 – UV spectrum of compound **2**

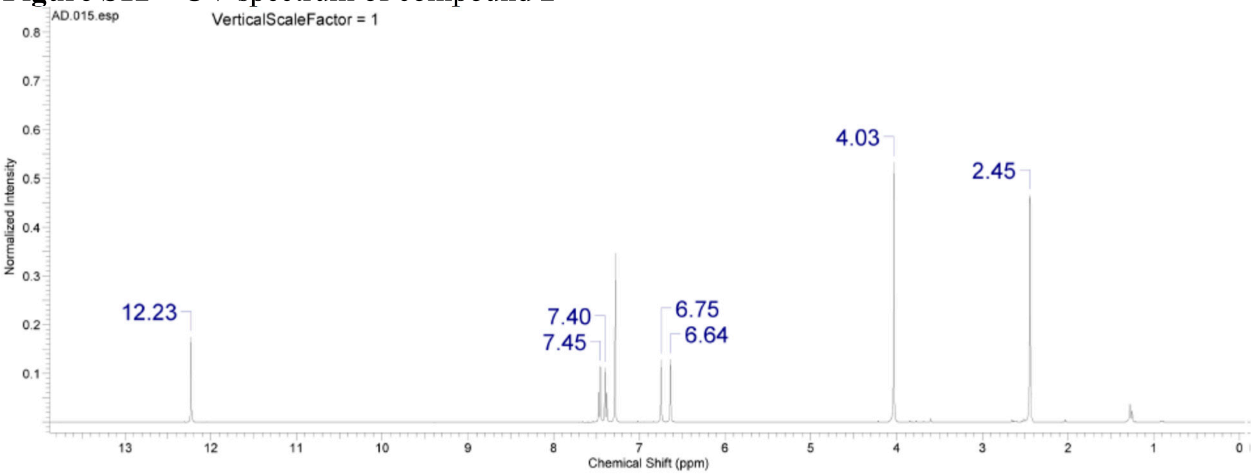

Figure S13 – ^{1}H NMR spectrum of compound **5**

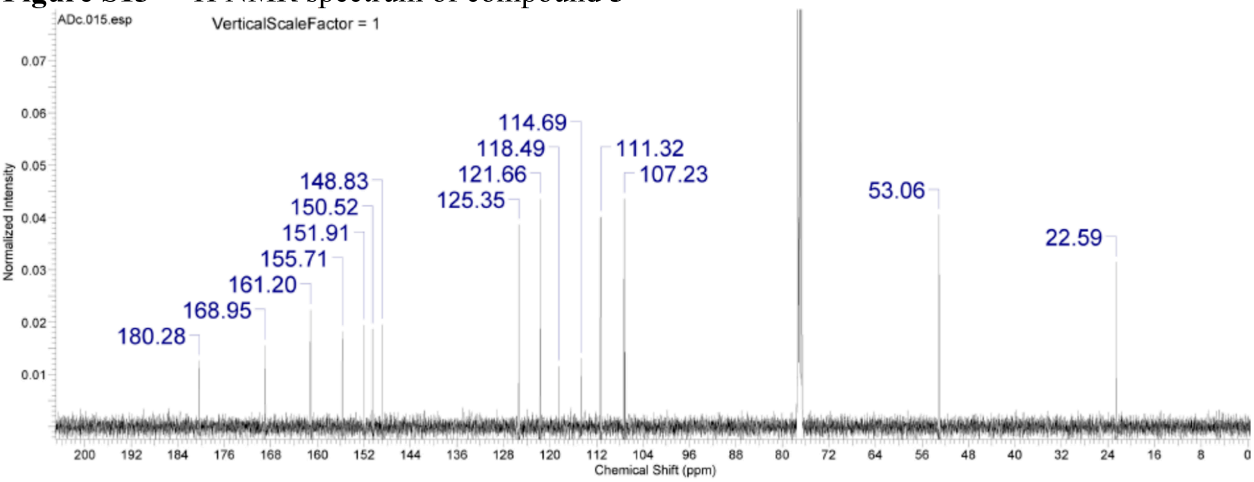

Figure S14 – ^{13}C NMR spectrum of compound **5**

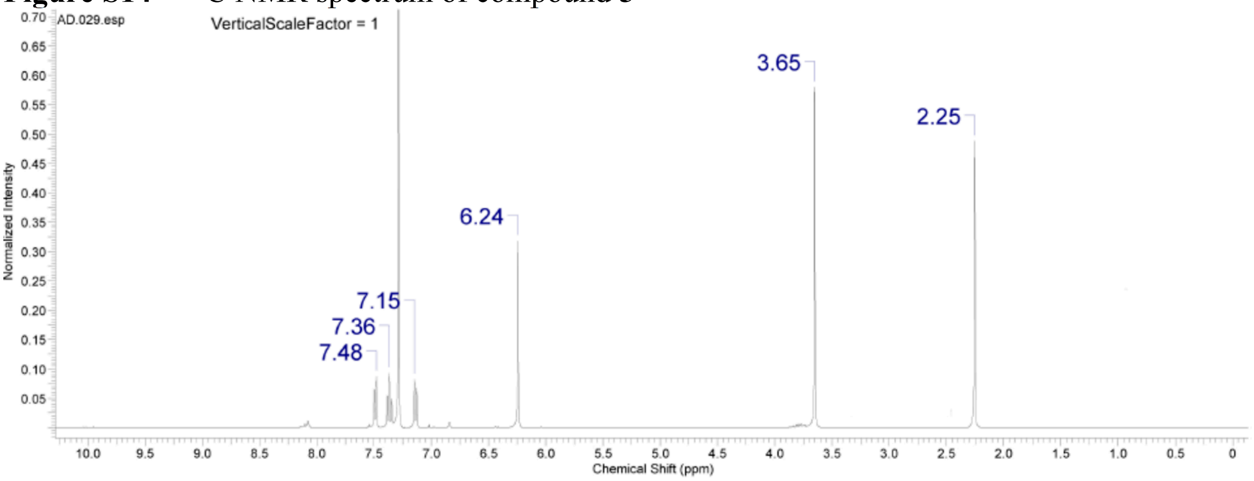

Figure S15 – ^{1}H NMR spectrum of compound **9**

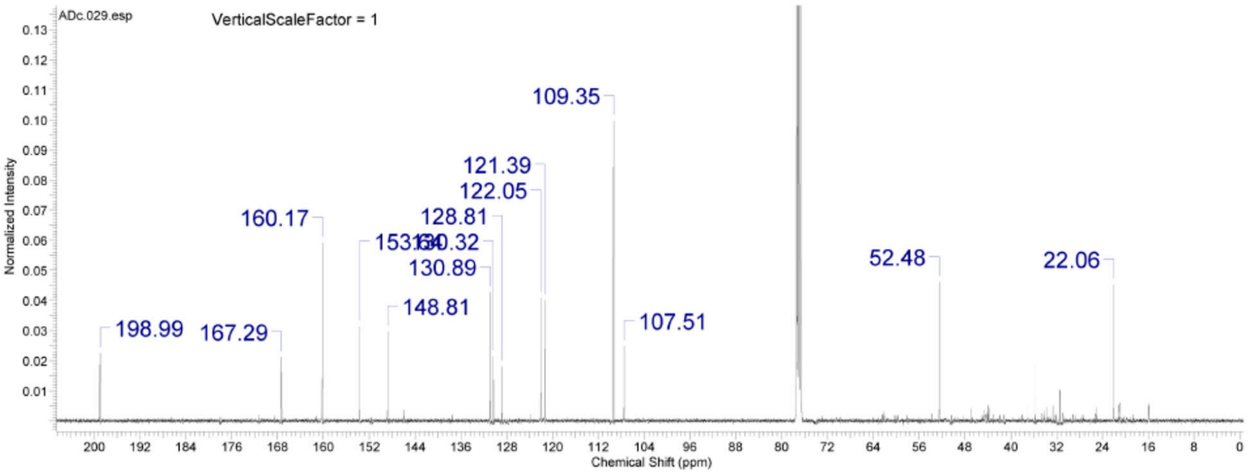

Figure S16 – ^{13}C NMR spectrum of compound **9**

Table S1. Crystal data and structure refinement for **1**

Empirical formula	$C_{16}H_{11}ClO_5$
Formula weight	318.70
Temperature, K	100(2)
Crystal system	monoclinic
Space group	$P2_1/c$
a,Å	7.1243(5)
b,Å	10.4999(7)
c,Å	18.0434(13)
β,°	92.221(6)
Volume, Å^3	1348.71(16)
Z	4
ρ_{calc}mg/mm^3	1.570
μ/mm^{-1}	2.730
F(000)	656.0
Crystal size, mm^3	0.21 × 0.18 × 0.13
Radiation	CuKα (λ = 1.54184)
2Θ range, °	9.748 to 139.516
Index ranges	$-8 \leq h \leq 5$, $-11 \leq k \leq 12$, $-21 \leq l \leq 21$
Reflections collected	6007
Independent reflections	2475 [R_{int} = 0.0823, R_{sigma} = 0.0520]
Data/restraints/parameters	2475/0/205
Goodness-of-fit on F^2	1.049
Final R indexes [I>=2σ (I)]	R_1 = 0.0600, wR_2 = 0.1685
Final R indexes [all data]	R_1 = 0.0674, wR_2 = 0.1835
Largest diff. peak/hole / e Å^{-3}	0.48/-0.58
$R_1 = \sigma||F_o| - |F_c||/ \sigma|F_o|$; $wR_2 = \{ \sigma[w(F_o^2 - F_c^2)^2]/ \sigma[w(F_o^2)^2]\}^{1/2}$; $w = 1/[\sigma^2(F_o^2)+(aP)^2 + bP]$, where $P = (F_o^2 + 2F_c^2)/3$; $s = \{ \sigma[w(F_o^2 - F_c^2)]/(n-p)\}^{1/2}$ where n is the number of reflections and p is the number of refinement parameters.	
